# Gate-Induced Static and Dynamic Nonlinearity Characteristics of Bilayer Graphene Field-Effect Transistors (Bi-GFETs)

**DOI:** 10.3390/mi16091031

**Published:** 2025-09-09

**Authors:** Varun Kumar Kakar, Munindra, Pankaj Kumar Pal

**Affiliations:** 1 Department of Electronics Engineering, National Institute of Technology, Srinagar Garhwal 246174, India; 2 Electronics & Communication Engineering Department, B.T. Kumaon Institute of Technology, Dwarahat 263653, India; 3 Department of Electronics & Communication Engineering, Shivalik College of Engineering, Dehradun 248001, India

**Keywords:** bilayer graphene field-effect transistor (Bi-GFETs), bilayer graphene, drain current characteristic, nonlinearity and scalability

## Abstract

In this study, the nonlinearity characteristics of bilayer graphene field-effect transistors (Bi-GFETs) are analyzed by using a small-signal equivalent circuit. The static nonlinearity is determined by applying mathematical operation on the drain current equation of Bi-GFETs. Furthermore, the closed expressions for the second- and third-order harmonic distortion (HD) and the intermodulation (IM) distortion of the second- and third-order for Bi-GFETs are analyzed graphically. Dynamic nonlinearity is studied and illustrated in the results by examining the input and output characteristics; i.e., the drain current versus the negative drain to the source voltage and the transfer characteristic curve at various gate voltages controlled by both the top gate as well as the back gate. The characteristic behavior of the gate voltage in Bi-GFETs at short channel lengths is observed and compared; that is, the characteristic curves exhibits strong nonlinearity, with a low power point with some kinks at high gate biasing and a constant linear region at low gate biasing. The quantitative values of the second-order harmonic distortion (HD) and intermodulation distortion (IM) of the proposed analytical model are −40 dB and −45 dB. Quantitative and qualitative outcomes of the characteristics of Bi-GFETs are compared with existing experimental data, which is available in the literature.

## 1. Introduction

Graphene has garnered significant attention as a potential material for analog/RF applications, owing to its exceptional electronic characteristics. Graphene exhibits higher carrier mobility and a superior saturation velocity, which can facilitate higher transconductance and enhance the device cut-off frequency in analog/RF applications. Further, the lack of a substantial bandgap open challenges the attainable ON–OFF current ratio, consequently constraining its application in digital logic. However, it has been reported in the literature that large-area monolayer, bilayer, and multilayer graphene have similar structures to transition metal dichalcogenides (TMDs), demonstrating tunable bandgaps [1,2,3]. Further, research indicates that laboratory-produced multilayer graphene, strategically stacked in a controlled manner, has emerged as an optimal candidate for the post-silicon semiconductor industries and fabrication foundries [4,5,6,7,8,9].

In contrast to traditional semiconductor materials, the tunable bandgap of bilayer graphene through an external electric field offers significant flexibility for the design and optimization of graphene field-effect transistors (GFETs) [10,11,12,13,14,15]. The electrically favorable characteristics of monolayer and bilayer graphene, controlled through moiré crystal orientation and twisted layers, present opportunities for high-frequency nanoelectronics devices [16,17,18]. The GFET based on monolayer graphene exhibits excellent electron transport mobility and efficient gate control [19], and the subsequent studies on bilayer graphene field-effect transistors (Bi-GFETs) demonstrate very high electron mobility and ballistic transport phenomena when subjected to an externally applied electric field, as demonstrated through gate biasing [16,20]. Further, tunable bilayer GFETs with low potential requirements for ultra-low voltage switching have also been explored using the Hamiltonian matrix’s non-equilibrium Green function (NEGF) formalism [21]. Evaluations based on balanced and unbalanced charge densities in Bi-GFETs reveal the fundamental physics of Bernal-stacked graphene layers, proposing the development of a basic logic inverter [22].

Models such as the semi-analytical Bi-GFET model [23] and the capacitance-based Bi-GFET model delve into the surface potential of both layers, translating it into the potential energy of a specific layer. The potential difference between the two layers is then employed to derive the output drain current [24]. The versatility of Bi-GFETs extends to various electronic applications, including low sub-threshold digital logic, high-voltage inverters, static random-access memory, back-gated Bi-GFETs, displacement electric field RF mixers with low losses, and dual-gate Bi-GFETs [25,26,27,28,29,30]. Most recent Bi-GFETs extends to various electronic applications sensors and photonic sensors Bi-GFETs [31,32].

The nonlinearity of short-channel GFETs is crucial for understanding their impact on transfer and output characteristics [33,34]. Previous GFET models have addressed static and RF performance nonlinearity, while traditional CMOS nonlinearity has been explored through mathematical operations on drain current [35,36,37]. However, existing Bi-GFET models have not sufficiently explained gate-induced nonlinearity of output and transfer characteristics with dual-gate control. In our proposed model, we modify the drain current model to account for layer potential and investigate both static and dynamic nonlinear characteristics of Bi-GFETs.

This study introduces a comprehensive nonlinearity model for Bi-GFETs organized into distinct sections. Section 2 describes the nonlinear models, specifically harmonic distortions (HDs) and intermodulation distortions (IMs), derived from the drain current using Taylor’s series. Section 3 encompasses the discussion of the results, including validation and comparison of the presented work, and Section 4 is the conclusion.

## 2. Nonlinearity Model of Bi-GFETs

**Figure 1:**
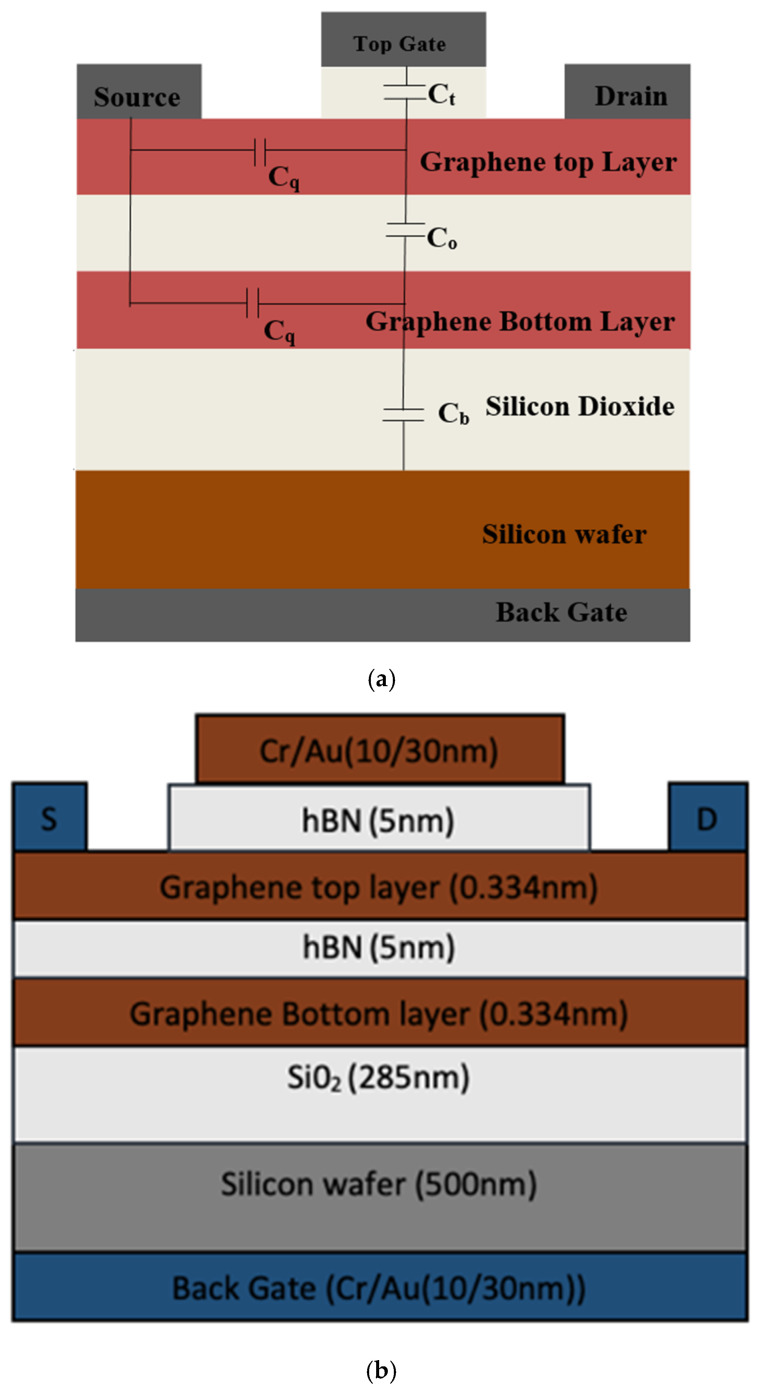
Basic block diagram of the proposed model Bi-GFET with the layers, quantum capacitance, and oxide capacitance shown in (**a**) and their thickness shown in (**b**).

The proposed model presenting static as well as dynamic nonlinear behavior of the Bi-GFET has been modeled for the device structure shown in Figure 1. The device structure is bilayer graphene with a high-k dielectric, hBN as the top oxide, and SiO_2_ as the back-gate oxide. The output drain current of Bi-GFETs is given in (6) as the function of the quantum capacitance (*C_q_*), top-gate voltage $V_{{GS}_{top}}$, back-gate voltage $V_{{GS}_{back}}$, and bilayer potential difference $V_{ch\left(n,p\right)}$.

### 2.1. Drain Current and Energy Bandgap Model

The drain current of a Bi-GFET is obtained with the help of the basic transport phenomenon of the charge carrier’s density of state (DOS) and quantum capacitance ($C_{q}$), etc. The DOS of bilayer graphene in (2) is directly connected to the energy bandgap ($E_{g}$), effective mass of charge carrier ($M_{eff}$), and energy level corresponding to the average vertical displacement electric field ($D_{AV}$). After biasing the top and bottom gates of Bi-GFETs, an opening bandgap is observed in bilayer graphene due to $D_{AV}$. This bandgap opening is explicitly explained with the help of an empirical relationship between the energy bandgap ($E_{g}$) and $\left(D_{AV}\right)$ in (1), where, ($V_{GSref}$) and ($V_{GS0}$) are the zero reference voltage applied to the top gate and the Dirac point offset voltage of the top gate, while, ($V_{BSref}$) and ($V_{BS0}$) are the zero reference voltage applied to the back gate and the Dirac point offset voltage of the back gate. $\varepsilon_{top}$, $\varepsilon_{box}$, $t_{top}$ and, $t_{box}$ are the top and bottom layer dielectric constants and dielectric thicknesses, respectively. The average vertical displacement electric field $\left(D_{AV}\right)$ can be formulated [28] as given in (1):

(1) $$D_{AV}=\frac{-\varepsilon_{top}(V_{GSref}-V_{GS0})}{t_{box}}+\frac{\varepsilon_{box}(V_{BSref}-V_{BS0})}{t_{box}}$$

However, the energy bandgap opening $E_{g}$ and $D_{AV}$ shows a relationship in (2) for the constant applied voltage at both gates to maintain a fixed value of $D_{AV}$ [28].

(2) $$DOS\left(E\right)=\frac{{2M}_{eff}}{\pi {\left(\hbar \right)}^{2}}\left\{K\left(E-\frac{E_{g}}{2}\right)+K\left(-E-\frac{E_{g}}{2}\right)\right\}$$

where ℏ represents the reduced Planck constant, *K* is the Heaviside step function, $M_{eff}=A*M_{e}$ is the effective mass, and *A* is the fitting parameter. Based on the DOS, the holes and electrons sheet densities, which work as channels in Bi-GFETs, are given as (4) and (6):

(3) $$p=\int_{-\infty }^{0}DOS\left(E\right)\left[1-f\left(E\right)\right]dE$$

(4) $$p=\frac{{2M}_{eff}}{\pi {\left(\hbar \right)}^{2}}{\left[E-K_{B}T\ln\left(exp\left(\frac{{E+E}_{F}}{K_{B}T}\right)+1\right)\right]}_{\left\{\frac{E_{g}}{2}\right\}}^{+\infty }$$

(5) $$n=\int_{0}^{+\infty }DOS\left(E\right)f\left(E\right)dE_{AV}$$

(6) $$n=\frac{{2M}_{eff}}{\pi {\left(\hbar \right)}^{2}}{\left[E-K_{B}T\ln\left(exp\left(\frac{{E-E}_{F}}{K_{B}T}\right)+1\right)\right]}_{\left\{\frac{E_{g}}{2}\right\}}^{+\infty }$$

These carrier concentrations can be used to calculate the total charge density $\rho_{gr}=q\left(p-n\right)$. This carrier concentration is used to find the charge density in bilayer grapheme [38]. Thus, we use the fundamental concept of the total charge equal to the product of capacitance and voltage, and so derivate the total charge density $\rho_{gr}$ with respect to the voltage along the graphene channel. This total charge density can be further used to calculate the quantum capacitance of the bilayer graphene ($C_{q}=\frac{d\;\rho_{gr}}{{d\;V}_{ch\left(n,p\right)}}$) as given in (8):

(7) $$\rho_{gr}=\frac{{2qM}_{eff}}{\pi {\left(\hbar \right)}^{2}}\left[-{qV}_{ch\left(n,p\right)}+\mathrm{sgn}\left(V_{ch\left(n,p\right)}\left(\frac{E_{g}}{2}\right)\right)\right]$$

(8) $$C_{q}=\frac{2{\left(q\right)}^{2}q\left|\Omega_{bl}\right|}{\pi {\left(\hbar \right)}^{2}}$$

where $\Omega_{bl}$ is the surface potential because of the scattering. This is the quantum capacitance of pure monolayer graphene. Since the DOS of monolayer graphene has died at the Dirac point, while for the bilayer graphene layer, the DOS is constant near the Dirac point due to dispersion [14], the modified quantum capacitance of bilayer graphene will be as given in (9):

(9) $${C^{\prime }}_{q}\left(E_{F}\right)=C_{q}*\frac{K_{B}T}{2\sqrt{2}}*\mathrm{erf}\left(¥,§\right)*Q\left(\alpha \right)+\frac{t_{1}}{2}*Q\left(¥,§\right)$$

where $\mathrm{erf}\left(¥,§\right)$ is the gaussian distribution error function, and in terms of the mathematical fitting parameters ¥ for holes, tends to negative infinity, and § for electrons, tends to positive infinity; $Q\left(\alpha \right)$ is the Fermi function in terms of α, a fitting parameter; $t_{1}$ is the vertical coupling parameter for the Bernal stack bilayer grapheme; and $Q\left(¥,§\right)$ is the Fermi function for $¥\;\left(holes\right)\;and\;§\;(electrons)$. This modified quantum capacitance provides the bilayer graphene channel potential $V_{ch(n,p)}$, which can be further utilized for the drain current calculation as given in (10) [39]:

(10) $$I_{ds}=\mu \left(n,p\right)W\frac{\mu \left(n,p\right)\int_{0}^{V_{ds}}\left|C_{q}V_{ch\left(n,p\right)}\right|dV+q\frac{n_{puddle}}{2}dV}{L+\mu \left(n,p\right)\int_{0}^{V_{ds}}\frac{1}{v_{sat(n,p)}}dV}$$

where *L* is the channel length, *μ* (*n*, *p*) is the carriers’ mobility in bilayer graphene, and *W* is the width of the Bi-GFET. $V_{ds}$ is the applied drain-to-source voltage and $C_{q}$ is the bilayer quantum capacitance. The rest of the parameters like $v_{sat(n,p)}$, $n_{puddle}$, and $V_{ch(n,p)}$ are explained in (11), (12), and (13), respectively. Saturation velocity of carriers can be represented as given in (11):

(11) $$v_{sat(n,p)}=\frac{\Omega }{V_{Ch}(n,p){\left(\pi ({q\;V}_{ch(n,p)}+n_{puddle})\right)}^{1/2}}$$

while the residual charge carriers are

(12) $$n_{puddle}=\frac{\Omega }{V_{Ch}(n,p){\left(\pi \rho_{gr}\right)}^{\frac{1}{2}+{A_{o}V(x)}^{2}}}$$

and the improved $V_{Ch}(n,p)$ channel voltage of the bilayer is given as (13) [28], the bilayer graphene channel voltage is the top-gate-applied voltage minus the *V*(*x*) channel potential, which varies from *x* = 0 to *L* (0 to $V_{DS}$), and the back-gate voltage minus the *V*(*x*) channel potential, which varies from *x* = 0 to *L* (0 to $V_{DS}$), with their corresponding dielectric capacitance $C_{top}\;and\;C_{box}$.

(13) $$V_{ch\left(n,p\right)}=\frac{\left[V_{{gs}_{top}}-V\left(x\right)\right]C_{top}+\left[V_{{bs}_{back}}-V\left(x\right)\right]C_{box}}{C_{top}+C_{box}+C_{q}}$$

### 2.2. Calculation of Harmonic and Intermodulation Distortion

Bi-GFETs exhibit nonlinearity in both static and dynamic states. To assess static nonlinearity, understanding higher-order harmonics is essential. In this study, we compute second-order and third-order harmonics using the Taylor’s series expansion of the drain current Equation (10), where $V_{GS}$ is the input voltage applied at the gate and source terminal of the Bi-GFET.

(14) $$I_{DS}={ℑ}_{1}V_{GS}+{ℑ}_{2}V_{GS}^{2}+{ℑ}_{3}V_{GS}^{3}+\cdots +{ℑ}_{n}V_{GS}^{n}$$

(15) $$I_{DS}=\frac{AB+AC{\left(\begin{array}{c} \frac{1}{C_{tox}+C_{box}+C_{o}+0.5\;C_{q}}{{(V}_{gs}}_{top}-\frac{V\left(x\right)}{2})C_{tox}-{{(V}_{gs}}_{back}-\frac{V\left(x\right)}{2})C_{box} \end{array}\right)}^{2}P\left[R\right]}{\left[\left(1+P-Q\right)\left(R\right)\right]}$$

where *A*, *B*, *C*, *P*, *Q*, and *R*, are fitting parameters and are the functions of drain voltage, effective mobility, mean free path, and channel length (*L*). Taylor’s series (${ℑ}_{1},{ℑ}_{2},{ℑ}_{3}$ coefficients are further used to formulate the HD and IM as follows: the “mean free path” and “fitting parameters A, B, C, P, Q, R” in the nonlinearity of the drain current are considered to keep the nonlinearity model compact and presentable, while all the fitting parameters are necessary in compact modeling. The drain current Equation (10) is a function of the channel voltage, saturation voltage, puddle charge, and channel length, while as per the nonlinearity modeling Equation (14), the Taylor’s series expansion is a function in terms of the overall gate voltage. The nonlinearity model is set to find HD and IM and so also finds the Taylor’s series coefficients; thus, Equation (15), in terms of the function of the channel voltage, saturation voltage, puddle charge, and channel length, is used to find the Taylor’s series coefficients, and so these fitting parameters are important in modeling:

(16) $${ℑ}_{1}=\left.\frac{\delta I_{DS}}{\delta V_{GS}}\right|=\frac{{{AC(2V}_{gs}}_{top}*C_{tox}^{2}+{{2V}_{bs}}_{back}*C_{box}^{2}-{{2V}_{gs}}_{top}*C_{tox}*{{2V}_{bs}}_{back}*C_{tox})P\left[R\right]}{C_{tox}+C_{box}{+C}_{o}+0.5\;C_{q}\left[\left(1+P-Q\right)\left(R\right)\right]}$$

(17) $${ℑ}_{2}=\left.\frac{\delta I_{DS}^{2}}{\delta V_{GS}^{2}}\right|=-\frac{\;A\;C\;(C_{tox}^{2}+C_{box}^{2}-{{2V}_{gs}}_{top}*C_{tox}*{{2V}_{bs}}_{back}*C_{tox})P\left[R\right]}{C_{tox}+C_{box}{+C}_{o}+0.5\;C_{q}\left[\left(1+P-Q\right)\left(R\right)\right]}$$

(18) $${ℑ}_{3}=\left.\frac{\delta I_{DS}^{3}}{\delta V_{GS}^{3}}\right|=\frac{4\;A\;C\;(C_{tox}*C_{tox})P\left[R\right]}{C_{tox}+C_{box}{+C}_{o}+0.5\;C_{q}\left[\left(1+P-Q\right)\left(R\right)\right]}$$

The second- and third-order harmonic distortions, *HD*_2_ and *HD*_3_, are derived using the coefficients from Taylor’s series outlined in Equations (19) and (20):

(19) $${HD}_{2}=\frac{1}{2}\left|\frac{{ℑ}_{2}}{{ℑ}_{1}}\right|V_{GS}=\frac{\left(C_{tox}^{2}+C_{box}^{2}-{{2V}_{gs}}_{top}*C_{tox}*{{2V}_{bs}}_{back}*C_{tox}\right)V_{GS}}{{{(2V}_{gs}}_{top}*C_{tox}^{2}+{{2V}_{bs}}_{back}*C_{box}^{2}-{{2V}_{gs}}_{top}*C_{tox}*{{2V}_{bs}}_{back}*C_{tox})}$$

(20) $${HD}_{3}=\frac{3}{4}\left|\frac{{ℑ}_{3}}{{ℑ}_{1}}\right|V_{GS}^{2}=\frac{3(C_{tox}*C_{tox})V_{GS}^{2}}{{{(2V}_{gs}}_{top}*C_{tox}^{2}+{{2V}_{bs}}_{back}*C_{box}^{2}-{{2V}_{gs}}_{top}*C_{tox}*{{2V}_{bs}}_{back}*C_{tox})}$$

Similarly, the second- and third-order intermodulation distortions, *IM*_2_ and *IM*_3_, are expressed in Equations (21) and (22):

(21) $${IM}_{2}=\frac{1}{2}\left|\frac{{ℑ}_{2}}{{ℑ}_{1}}\right|V_{GS}=\frac{\left(C_{tox}^{2}+C_{box}^{2}-{{2V}_{gs}}_{top}*C_{tox}*{{2V}_{bs}}_{back}*C_{tox}\right)V_{GS}}{{{(2V}_{gs}}_{top}*C_{tox}^{2}+{{2V}_{bs}}_{back}*C_{box}^{2}-{{2V}_{gs}}_{top}*C_{tox}*{{2V}_{bs}}_{back}*C_{tox})}$$

(22) $${IM}_{3}=\frac{3}{4}\left|\frac{{ℑ}_{3}}{{ℑ}_{1}}\right|V_{GS}^{2}=\frac{3(C_{tox}*C_{tox})V_{GS}^{2}}{{{(2V}_{gs}}_{top}*C_{tox}^{2}+{{2V}_{bs}}_{back}*C_{box}^{2}-{{2V}_{gs}}_{top}*C_{tox}*{{2V}_{bs}}_{back}*C_{tox})}$$

The quantum capacitance ($C_{q}$) of the bilayer graphene as given in (8) depends on the surface potential of bilayer graphene $\left|\Omega_{bl}\right|$. The drain current is calculated using $C_{q}$ and then the modified drain current is used to find the HD and IM for the Bi-GFETs.

## 3. Results and Discussion

The proposed model has shown comparable transfer and output characteristics with and without biasing the back gate. The application of gate potential induces an electric field on the vertical side of the A-B Bernal-stacked bilayer graphene, allowing for the tuning of a finite small bandgap. The introduction of graded doping to the bilayer graphene enables control over the diffusion of metal contact-induced source (S)/drain (D) doping. This controlled doping mechanism is reflected in improved transfer characteristics for the proposed device, namely the bilayer graphene field-effect transistor (Bi-GFET). The analytical model of the proposed Bi-GFET is rigorously validated through a comprehensive comparison with TCAD-based simulations. The validation process incorporates the density gradient quantum model, Shockley–Read–Hall recombination, concentration and electric-field-dependent Lombardi model, auger recombination, and bandgap-narrowing model. This ensures a robust assessment of the proposed Bi-GFET’s performance by considering a range of essential factors and phenomena.

The short-channel effects like contact resistance of the bilayer graphene device can be minimized by introducing thin interfacial layers between the metal contacts and the grapheme channel, while graphene can improve contact resistance. For example, as-grown graphene has shown better adhesion and lower contact resistance compared to transferred graphene [15]. The transient and AC frequency responses of the bilayer graphene device exhibit unique transient and AC frequency responses due to their special electronic band structure. Transient measurements reveal how the device reacts to sudden changes in voltage or current, while the AC frequency response studies how the device reacts to oscillating signals. These responses are crucial for understanding and optimizing the performance of graphene-based electronics. In monolayer graphene field-effect transistors (GFETs), increased self-heating under pulsed measurements leads to higher resistance and lower current compared to DC measurements. However, in bilayer graphene, the opposite effect is observed, where pulsed measurements show higher current levels than DC measurements, suggesting a different self-heating mechanism or other transient effects [40]. Harmonic distortion in bilayer graphene devices operating at high frequencies arises from the device’s inherent nonlinearity, meaning that the output signal is not a perfect replica of the input signal. This distortion manifests as the generation of harmonics, which are signals at integer multiples of the fundamental frequency. Analyzing and estimating these harmonic components is crucial for understanding and optimizing device performance, especially in applications where linearity is critical, such as RF and microwave electronics. Various techniques are used to estimate the error introduced by harmonic distortion:

- Direct Measurement: Measuring the amplitude and phase of the fundamental and harmonic components using spectrum analyzers or network analyzers.
- Simulation: Employing circuit simulators to model the device and predict its harmonic response.
- Analytical Models: Developing physics-based models of the device to predict its nonlinear behavior and harmonic generation.

To compare and validate the proposed model, Bi-GFETs are fabricated on Si/SiO_2_ substrate with Ni contact and Al_2_O_3_ (HfO_2_) high-k for the input/transfer characteristic as the top-gate dielectric with Ni/Ti top electrodes. For studying the output characteristics, Al_2_O_3_ is taken as the top-gate dielectric and the contact-induced doping in the S/D region is considered as shown in Figure 1. For the output characteristics, intrinsic bilayer graphene has been sandwiched between the top gate and back gate with high-k dielectric materials. The vertical electric field control and tuning of the Bi-GFET is achieved by applying back-gate voltage (V_BG_), which ranges in this from −20 V to −90 V. Drain current characteristics and equations are both useful to explain the nonlinearity of the Bi-GFET model.

**Figure 2:**
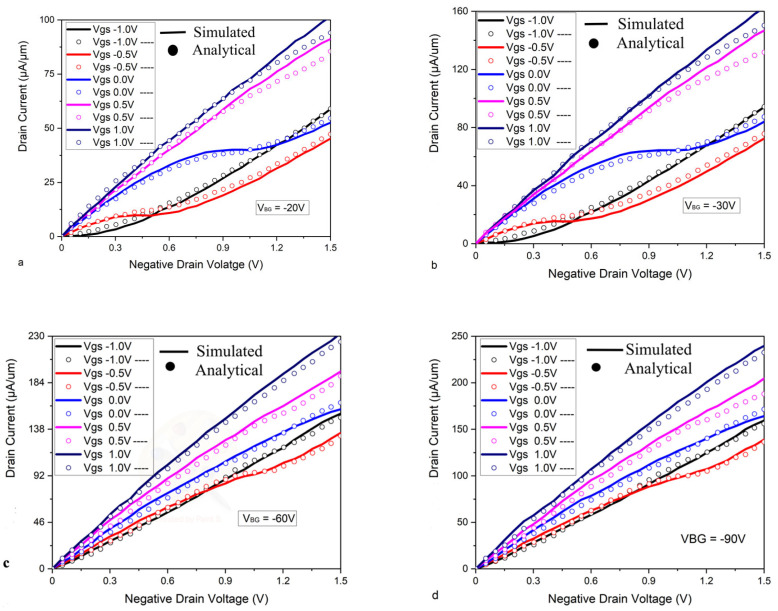
The nonlinear output characteristics of Bi-GFET with back-gate contact-induced control and dual-gate vertical electric field effect have been shown at various back-gate voltages. (**a**) shows the negative output characteristic curve at = −20 V and illustrates the nonlinear line of drain current over a range of top-gate voltage (−1.0 V to 1.0 V). (**b**–**d**) illustrate the negative characteristic curve at = −30 V, −60, −90 V respectively and represent the nonlinear characteristic behavior over a range of top-gate current (−1.0 V to 1.0 V) [25,28].

**Figure 3:**
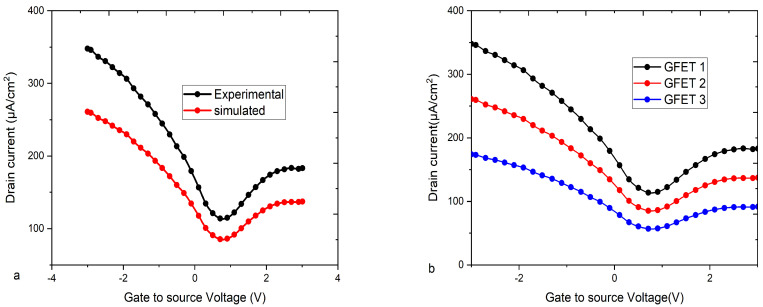
(**a**) Simulation model calibration against the experimental GFET. (**b**) shows the transfer characteristic; i.e., the change in the drain current with different ON/OFF ratios of the current conduction.

The drain current nonlinearity and scalability have been studied to a wide range of $V_{BG}$ (−20 V to −90 V), with input-gate voltage ranging from −1.0 V to 1.0 V, thus showing a great dual-gate control and bandgap tunable effect of bilayer graphene by the vertical electric field provided by biasing both gates. For low back-gate voltages, the nonlinear behavior of the drain current over the range of the drain voltage is shown in Figure 2. As the back-gate voltage is increased to a higher value, the drain current shows the nonlinearlity, as shown in Figure 2a. The drain current behaves more nonlinearly at low voltage top-gate biasing than it does for the same back-gate voltage biasing. The scalable drain current increases from 160 µA/µm to 250 µA/µm, as shown in Figure 2a–d concerning the increment of the $V_{BG}$ from −20 V to a higher value. Thus, the proposed Bi-GFET model presents nonlinearity and scalability. The proposed model has been calibrated with experimental work from [41]. The proposed model has shown close values of drain current as shown in Figure 3a. Comparison of the proposed model is illustrated in Figure 3b, which proves that the proposed model is superior in comparison to [41,42] with a different current ON/OFF ratio.

The high nonlinearity observed at the gate bias corresponding to the peak cut-off frequency is attributed to the strong modulation of charge carrier concentration and transconductance at this bias point. Around the peak cut-off frequency, the device operates in a highly dynamic regime where small changes in gate voltage result in significant variations in current, leading to enhanced nonlinear effects. In contrast, before and after this gate bias, the transconductance variation is relatively moderate, resulting in reduced nonlinear distortion.

**Figure 4:**
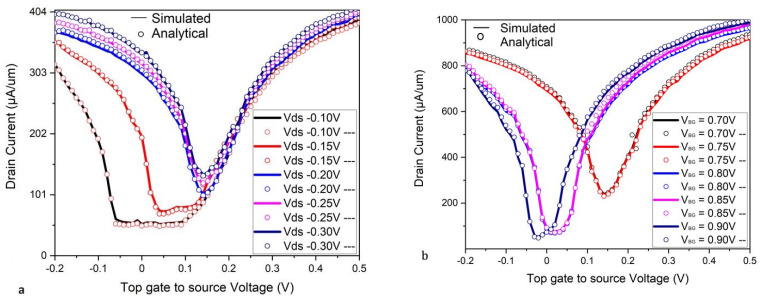
The nonlinear transfer characteristics of the Bi-GFET with the back-gate contact-induced control and dual-gate vertical electric field effect have been shown at various back-gate voltages and top-gate drain voltages. (**a**,**b**) illustrate the nonlinear nature of the transfer characteristic of the Bi-GFET at top-gate drain voltages over a range (−0.1 V to −0.3 V) and at back-gate voltages over a range (0.7 V to 0.9 V) [28].

The doping has also shown the ambipolar nature of Bi-GFET; as shown in Figure 4a, the transfer characteristic curves of the proposed model depicts the increasing ambipolarity. To explore the ambipolar nature, as well as the effect of the drain voltage observed with the enhanced control of top gate, the Bi-GFET is simulated at five different drains to the source voltage ($V_{ds}$ = 0.1, 0.15, 0.2 V, 0.25 V, and 0.3 V) as shown in Figure 4a. The nonlinear transfer characteristics of the Bi-GFET with the back-gate contact-induced control and dual-gate vertical electric field effect have been shown at various back-gate voltage in Figure 4b. When $V_{ds}$ is increased, ambipolarity of the input curve also increases. Initially, a back gate biased ($V_{bs}$) at a very low back-gate voltage (0.71 V) is analytically calculated with the measured data for the transfer characteristic and simulated at the TCAD tool for validation.

**Figure 5:**
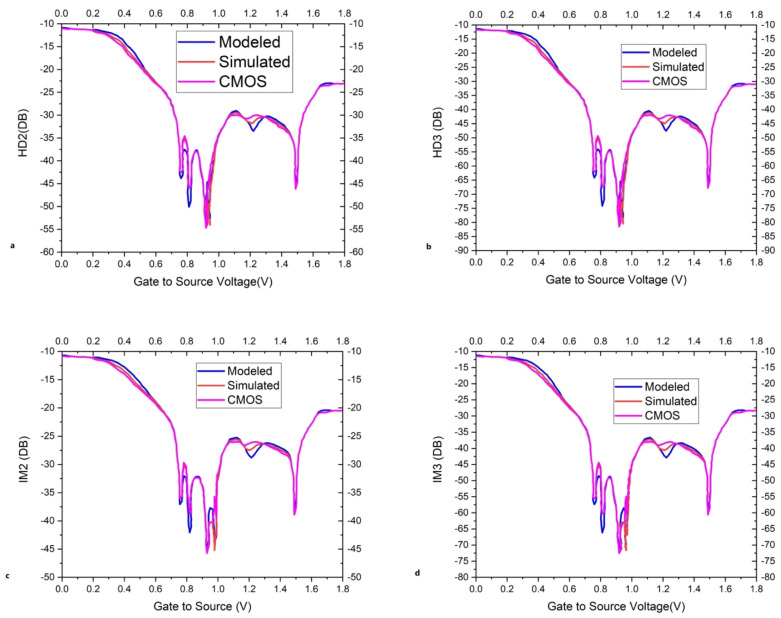
HD_2_, HD_3_, IM_2_, and IM_3_ harmonic distortion and intermodulation distortion of the proposed analytical model are compared with the simulated model of the second order and the third order in (**a**), (**b**), (**c**), and (**d**), respectively.

The measured and simulated data have shown good agreement with the available literature and have less than 10% normalized root mean square (NRMS) error between the measured and simulated data. The transfer characteristic curve of Bi-GFETs shows a high linearity with a gradual increase in the drain-to-source voltage biasing. Harmonic distortion (HD) and intermodulation distortion (IM) of the proposed analytical model are compared with the simulated model and conventional MOSFET of the second order and the third order in Figure 5. HDs of the second order with varying top-gate voltages are presented in Figure 5a. HDs of the third order with varying top-gate voltages are presented in Figure 5b. HDs of the third order are low in comparison to the second order. HD of the proposed analytical model and simulated model are very much similar while conventional MOS has indifference values. Intermodulation distortions of the second order with varying top-gate voltages are presented in Figure 5c. Intermodulation distortions of the third order with varying top-gate voltages are given in Figure 5d.

The nonlinear analysis of the proposed Bi-GFETs was carried out statistically, and the potential RF performance of the proposed model at 30 GHz frequency is given in Table 1 to compare with the available MOS, CMOS, and CNTFETs technologies. The proposed model has shown better RF performance [35] than the conventional MOS, CMOS, and CNTFETs [36]. Analytical results of the proposed Bi-GFETs model at two different channel lengths (L) are given in the below Table 1. Table 1 presents and compares the RF performance loss of the GFETs at different channel lengths and frequencies, as well as the input intercept point of the third order (IIP3) and total gain compression with the available literature. The nonlinearity effect influences the Bi-GFET’s performance by the addition of harmonic and intermodulation distortions, lowering the gain of the GFETs, shifts in the DC offsets, and cross modulation of the AM/PM (amplitude and pulse modulation), etc.

## 4. Conclusions

The proposed Bi-GFET model has been analyzed for the characteristics and nonlinearity of the Bi-GFET at various scaling channel lengths for the dual-gate biasing with fixed DC conditions. An explicit static nonlinearity model, the RF performance, and the dynamic nonlinearity of the transfer and input/output drain current have also been presented in this study. The significance of second- and third-order distortion terms were investigated for top-gate biasing and bottom-gate biasing with fixed DC-biasing conditions. High nonlinearity is observed at the gate biasing corresponding to the peak cut-off frequency and low nonlinearity is observed before and after this particular gate biasing. Table 1 shows the comparison of the Bi-GFET’s nonlinearity with a CNTFET and a MOSFET, which shows the low losses at short channel lengths for Bi-GFET. It has been observed that the high intermodulation loss expected in Bi-GFET is absent, which is because of a nonlinear dispersive band of bilayer graphene.

## Figures and Tables

**Table 1:** Comparison of the IIP3 and conversion loss of the proposed model Bi-GFETs with a channel length of *L* with the CMOS, MOSFET, and CNTFET mentioned by reference.

Operating Frequency [Ref.]	IIP3 (dBm)	Conversion Loss (dB)	*L* (µm)
10 MHz [43]	13.8	~30 to 40 (~38.89)	2
30 GHz [44]	12.8	19	0.5
NA [45]	4.9	20–22 (~21.10)	1
NA [46]	22	~15	0.25
NA [47]	27	10	2
2 GHz [48]	19	5	0.75
4.3 GHz [49]	30	10	0.25
300 MHz [50]	20	15	0.5
30 GHz [proposed with back gate unbiased]	11.92	17	2.4
40 GHz [proposed with back gate biased]	10.88	11.28	0.44

## Data Availability

The original contributions presented in this study are included in the article. Further inquiries can be directed to the corresponding author.
